# Gender Differences for His Bundle Pacing Long-Term Performance in the Elderly Population

**DOI:** 10.3390/jcdd12030088

**Published:** 2025-02-26

**Authors:** Catalin Pestrea, Ecaterina Cicala, Dragos Lovin, Adrian Gheorghe, Florin Ortan, Rosana Manea

**Affiliations:** 1 Department of Interventional Cardiology, Clinical County Emergency Hospital of Brasov, 500326 Brasov, Romania; cicalaecaterina@gmail.com (E.C.); dlovin94@gmail.com (D.L.); gheorghe.adrian9393@gmail.com (A.G.); ortan.florin@gmail.com (F.O.); 2 Faculty of Medicine, “Transilvania” University of Brasov, 500019 Brasov, Romania; rosanamanea@gmail.com; 3 Department of Radiology and Medical Imaging, Clinical County Emergency Hospital of Brasov, 500326 Brasov, Romania

**Keywords:** His bundle pacing, feasibility, elderly, long-term follow-up, gender differences

## Abstract

Background and aims: His bundle pacing (HBP) is considered the most physiological form of cardiac pacing. Although feasibility studies have included older patients, specific data for HBP in this population are scarce. This study aimed to evaluate gender differences in HBP long-term performance in elderly patients with atrioventricular (AV) block. Methods: This retrospective study included 73 patients aged over 65 years with successful HBP and at least 2 years of follow-up. The patients’ baseline and follow-up clinical and procedural characteristics were recorded. Results: The mean age of the cohort was 72.8 ± 6.3 years, with 43 males and 30 females. The paced QRS complex was significantly narrower than the baseline value for both genders. Females had a narrower-paced QRS complex without differences in detection, type of His bundle capture, impedance, or fluoroscopy time. The pacing threshold increased progressively, reaching statistical significance compared to the baseline values at the two-year follow-up. The pacing threshold increased by more than 1 V over the follow-up period in twenty-four patients (32.9%) and by more than 2 V in six patients (8.2%), with no significant difference between genders. The pacing threshold increase occurred within the first year for most patients, without gender differences. Multivariate Cox regression analysis demonstrated that the paced QRS duration, left ventricular ejection fraction, and ischemic cardiomyopathy were significantly associated with the pacing threshold increase over time. Conclusion: In elderly patients with AV block, HBP remains a feasible pacing method, without significant gender differences, over a long-term follow-up period. Pacing threshold increases are expected in up to one-third of the patients, requiring regular follow-ups to adjust the programmed parameters and optimize battery longevity.

## 1. Introduction

Cardiac pacing is a life-saving procedure performed increasingly worldwide. The longer life expectancy in the general population due to better healthcare is partly responsible for this constantly growing number of devices implanted [1]. Consequently, the mean age of the patients subjected to pacemaker implantation has increased in the last decades. In a study by Matsubara et al., the mean age at initial pacemaker implantation increased by 12.1 years over the last 50 years, with the most significant increase in patients over 70 years [2]. Furthermore, several studies looked at gender differences in device implantation, showing that males had a higher prevalence of atrioventricular disturbances and higher long-term mortality after the procedure [3]. In contrast, females were older at the time of the procedure and had a higher periprocedural morbidity [4].

Increasing evidence showed that conventional right ventricular pacing induces electrical and mechanical dyssynchrony. This translated into decreased left ventricular performance in a significant proportion of patients [5].

Consequently, almost two decades ago, His bundle pacing (HBP) was implemented as a more physiological pacing technique [6]. With time, several disadvantages, such as difficulty and procedural parameters, determined a shift towards left bundle branch area pacing (LBBAP), a more reproducible physiological pacing method with superior pacing parameters [7]. Nevertheless, in successful HBP cases, all studies have shown net clinical benefit in cardiac function and mortality, both for bradycardia and cardiac resynchronization indications [8,9]. Therefore, if not as a primary goal, HBP can still be useful as an alternative to failed LBBAP.

Although many observational HBP studies included elderly patients (65 years old and over) in their study groups, specific results for this category are scarce, especially in the long term. We have previously shown that the procedure was feasible in the elderly and the very elderly (over 80 years old), but gender-specific data are missing for these patients [10].

With the initial hypothesis that there are no significant differences, this study aimed to evaluate and compare gender-specific outcomes in HBP long-term performance in elderly patients with atrioventricular (AV) block.

## 2. Materials and Methods

### 2.1. Study Design

This was a retrospective, analytical, single-center study.

### 2.2. Patient Selection

All patients over 65 who underwent permanent HBP for second or third-degree AV block between August 2018 and December 2021 in the Cardiac Pacing Laboratory of the Brașov County Clinical Emergency Hospital in Romania were reviewed for inclusion in the study. Only patients with intraprocedural electrophysiological criteria for His bundle (HB) capture and a follow-up period of at least 2 years were accepted. In the end, 73 patients were included in the analysis. The baseline demographic and clinical characteristics of the patients were recorded.

### 2.3. Pacing Procedure

The physiological pacing procedure was performed using the C315 His catheter (Medtronic, Minneapolis, MN, USA) with the Select Secure 3830 lead (Medtronic, Minneapolis, MN, USA). The catheter was placed under fluoroscopic guidance at the superior part of the tricuspid valve, where unipolar mapping identified the HB signal. Pacing at decremental amplitude was performed to evaluate HB capture. In patients with a baseline narrow QRS complex, selective HBP was defined as a paced QRS complex and an ST-T interval identical to the baseline morphologies. Non-selective HBP was defined as a lack of an isoelectric line after the pacing artifact, with a “pseudo-delta” aspect at the beginning of the QRS complex and a transition with decremental pacing amplitude from non-selective HBP to either selective HBP or pure myocardial capture [11].

In patients with a baseline-wide QRS complex, the same criteria for selective/non-selective were applied as above, adding the correction of bundle-branch block if the paced QRS complex duration was lower than 130 ms. No ventricular back-up leads were implanted.

The pacing and sensing thresholds and the fluoroscopy time were recorded.

### 2.4. Follow-Up

The patients were followed in the outpatient clinic at 1, 3, 6, and 12 months after the procedure and then yearly. Sensing and pacing thresholds, as well as late complications, were recorded during follow-up. At each device interrogation, the pacing output was set at 1 V above the HB capture threshold.

### 2.5. Statistical Analysis

Continuous variables were presented as mean ± one standard deviation. Categorical variables were presented as frequencies and percentages. A statistical comparison of means was performed using the t-test or the Mann–Whitney U test for independent groups and the t-test or Wilcoxon test for dependent groups according to the normality of distribution. The Chi-squared test evaluated the statistical difference between percentages. The Kaplan–Meier survival curve and the log-rank test were used to estimate event-free survival in the different pacing groups. Univariate and multivariate Cox proportional hazards regression analyses were performed to investigate the potential risk factors of pacing threshold increase during follow-up. A confidence interval of 95% was used for all tests, and a *p* < 0.05 was considered statistically significant.

Statistical analysis was performed using SPSS software version 26.0 (IBM, Armonk, NY, USA).

### 2.6. Ethical Considerations

The study complied with all aspects of the Declaration of Helsinki and was approved by the institutional ethics committee.

All patients provided written informed consent before the procedure.

## 3. Results

The mean age of the cohort was 72.8 ± 6.3 years, with 43 males and 30 females. Table 1 presents the baseline characteristics of the entire cohort and for each gender.

The two groups had no significant difference regarding baseline QRS duration and morphology, ejection fraction, comorbidities, or medical treatment taken. The only significant difference was a larger left atrial diameter recorded in the male group.

### 3.1. Procedural Characteristics

All the procedural characteristics are presented in Table 2. The paced QRS complex was significantly narrower than the baseline value (*p* < 0.001 overall and for each gender group). The paced QRS duration was 98.9 ± 20.1 ms in males and 88.1 ± 16.6 ms in females (*p* = 0.02). There were no significant differences in detection times (*p* = 0.94), His bundle capture type (*p* = 0.81), impedance (*p* = 0.79), or fluoroscopy time (*p* = 0.95).

### 3.2. Follow-Up

All patients reached the two-year follow-up, 63 patients the three-year follow-up, 33 patients the four-year follow-up, and 14 patients the five-year follow-up. There was one case of pocket infection that required reintervention and one case of a large pericardial effusion drained percutaneously without recurrences. No other complications, including lead dislodgements, that led to pacing interruption were noted.

Over the follow-up period, thirteen patients (17.8%) became completely pacemaker-dependent without an underlying escape rhythm. In the rest, the R wave sensing showed a non-statistically significant difference compared to the baseline (3.47 ± 2.35 mV vs. 3.81 ± 2.41 mV, *p* = 0.24).

The pacing threshold increased by more than 1 V over the follow-up period in twenty-four patients (32.9%, fifteen males and nine females, *p* = 0.80) and by more than 2 V in six patients (8.2%, four males and two females, *p* = 1). None of the patients had complete loss of HB capture. The 1 V increase occurred for sixteen patients (21.9%) in the first year of follow-up. For four patients (5.5%), there was an increase between the first and the second year, and for four patients (5.5%) after two years, without differences between genders.

**Figure 1:**
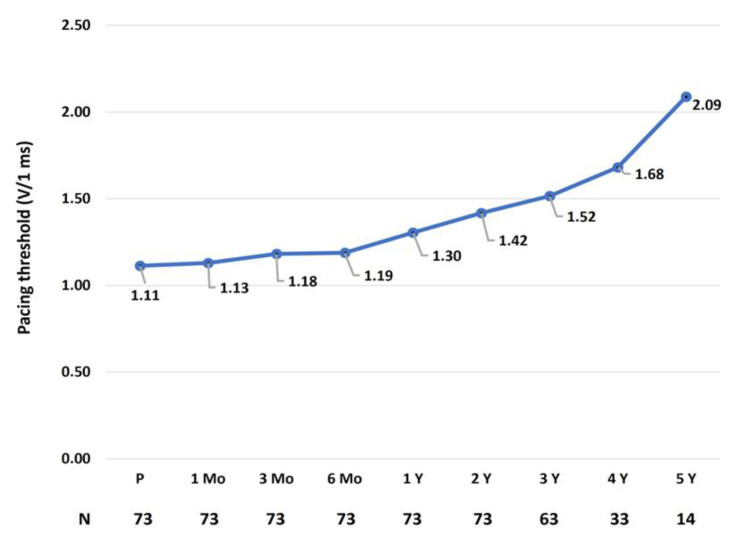
Mean pacing thresholds (V at 1 msec pulse duration) for the entire cohort at each visit. N, number of patients; P, procedural; Mo, months; Y, years.

The mean pacing thresholds at each visit are presented in Figure 1.

As illustrated in the figure, the pacing threshold increased progressively, reaching statistical significance compared to the baseline values at the two-year follow-up (*p* = 0.004).

**Figure 2:**
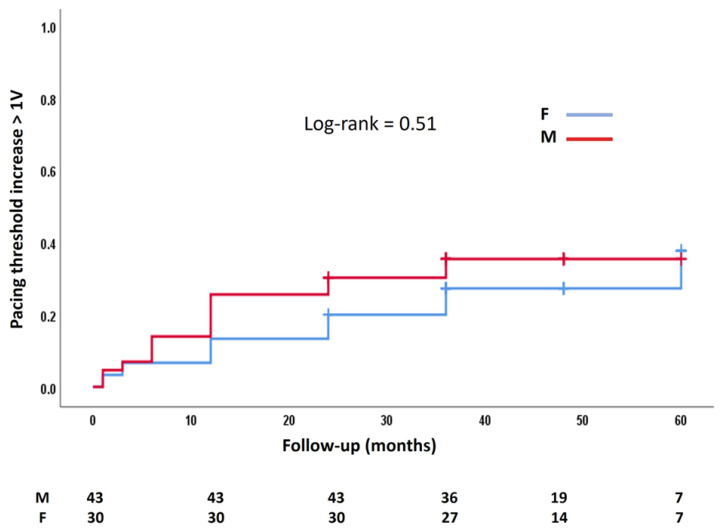
The Kaplan–Meier curve compares the pacing threshold increase of more than 1 V over time between gender groups. M, male; F, female.

There was no significant difference between genders in the occurrence of pacing threshold increases by more than 1 V during follow-up (Figure 2).

Univariate Cox regression analysis of all possible risk factors demonstrated that the paced QRS duration, the left ventricular ejection fraction, and ischemic cardiomyopathy were significantly associated with pacing threshold increase over time (Table 3). The same association was also confirmed by a subsequent multivariate Cox regression model in which we introduced all variables with a *p* < 0.1 in the univariate analyses.

At the last follow-up, most patients maintained the initial type of HB capture and the transition sequence at decremental pacing (32 non-selective HB to myocardial capture and 29 non-selective to selective HB capture). Five patients who initially transitioned from non-selective to myocardial capture developed a transition from non-selective to selective HB capture. Six patients who transitioned initially from non-selective to selective switched to a transition from non-selective HB to myocardial capture (Table 4). No significant differences in these transitions were recorded between genders.

## 4. Discussion

The main findings of this study were as follows: (i) in the elderly population, HBP is a feasible pacing technique without significant long-term associated complications that lead to pacing failure; (ii) a pacing threshold increase of up to 2 V occurred in approximately a third of the cohort, but of more than 2 V in only 8% of the patients; (iii) there were no significant differences between genders for HBP performance both during the procedure and in the long-term.

HBP is supported by the strong physiological argument that it uses the entire infra-nodal conduction system to provide fast and synchronous biventricular activation. This explains the short QRS duration after HBP recorded in our cohort, which supports the existing data that HBP is associated with the narrowest paced QRS complex [12]. Also, it has been shown that HBP can correct infra-nodal conduction abnormalities, generating a narrower QRS complex than the baseline value [13].

An important issue related to HBP is the higher procedural pacing threshold compared to conventional right ventricular pacing [14]. This could be explained anatomically by a deeper position in the septum and/or the fibrous sheath encapsulating the HB [15]. Nevertheless, initial studies have shown that the procedural pacing threshold depends on operator experience, with a trend of significant improvement during the learning curve [16]. The same argument applies to fluoroscopy times. In this regard, our procedural pacing thresholds of around 1 V at 1 ms pulse duration and fluoroscopy times of around 9 min align with the results published by other experienced and high-volume centers [17].

One of the main reasons for a shift towards LBBAP in laboratories worldwide is the risk of pacing threshold increase over time during HBP. Zanon et al. showed in a multicenter observational study that almost 25% of the patients had a follow-up threshold of more than 2.5 V, and 6% reached a threshold of more than 3.5 V [18]. On the other hand, Vijayaraman et al. reported that in 143 patients with AV block and HBP, there was a pacing threshold increase of more than 1 V in 5.6% over a two-year follow-up [17]. Possible mechanisms for the pacing threshold increase are lead slack and orientation to the HB, micro-dislodgements, local fibrosis, and the progression of conduction system disease [19]. In our data, although a third of the patients had a pacing threshold increase with more than 1 V, only 8.2% increased with more than 2 V. Moreover, the pacing threshold increase occurred mostly in the first year after the procedure, confirming the trend described in previous studies [19].

Because the HB lead is proximally placed in the conduction system, there is the concern of distal progression of the conduction disease, making HBP redundant. Therefore, in our population, these results and the fact that no patient completely lost the HB capture represent arguments against such significant distal progression.

Additionally, the pattern of HB capture during the procedure and at follow-up proves lead stability and lack of conduction system disease worsening, with 83.6% of the patients maintaining their initial transitions over time.

Previous studies looked at sex-based differences in outcomes in patients with HBP. For example, Wu et al. showed that females with left bundle branch block derive more benefits from HBP, with cardiac size and QRS duration contributing partially to these sex-based variations [20]. Also, Stangl et al. found no significant gender-based differences in the success of HBP implantations, measuring the threshold at implantation, one day after, and four weeks after implantation between 22 females and 39 males [21].

To our knowledge, this was the first study to evaluate gender differences in HBP performance over a mid- to long-term follow-up period in patients with AV block. Our results showed no significant difference in both acute results and the evolution of the pacing threshold values. Both genders displayed a constant trend of pacing threshold increases over time without reaching values that would necessitate lead revision. This finding is important since, in the elderly population (especially the very elderly), the decrease in battery longevity associated with higher pacing thresholds may not be a significant issue due to a lower life expectancy in this category.

An important finding was that the procedural pacing threshold was not a predictive factor for pacing threshold increase. This has been also shown in previous studies. Vijayaraman et al. found no association between clinical and procedural characteristics, except for the lead slack and pacing threshold increase over time in 294 HBP patients [19]. The same observation was made by Upadhyay et al. in 140 patients, proving that only a history of atrial fibrillation was predictive of pacing threshold increase [22]. Our observation that paced QRS duration was significantly associated with a subsequent rise in pacing thresholds could be explained by the more diffuse conduction system disease in patients with a wider paced QRS complex.

Given the long follow-up period, we believe the key message of this study is that irrespective of gender, in elderly patients, HBP is still a viable option for physiological pacing, either as a first attempt or as a bailout after a failed attempt at left bundle branch area pacing.

Nevertheless, several limitations of the study should be mentioned. This was a retrospective, single-center study with a relatively small number of patients. Although the study included a theoretical follow-up of up to five years, only 14 patients were observed for this duration, limiting conclusions regarding long-term HBP stability. Future studies should aim for a more balanced follow-up duration across all participants. The procedures were performed using only one type of delivery system. With the current technological advances, the results may have been different. Finally, this was a procedural feasibility study that did not assess the clinical impact of HBP in the study group in the long term.

## 5. Conclusions

In elderly patients with AV block, HBP remains a feasible pacing method, without significant gender differences, over a long-term follow-up period. Pacing threshold increases are expected in up to one-third of the patients, requiring regular follow-ups to adjust the programmed parameters and optimize battery longevity.

## Figures and Tables

**Table 1:** Baseline patient characteristics.

Baseline Characteristics	All	Male	Female	*p*
Number of patients	73	43	30	
Age (years, mean ± SD)	72.8 ± 6.3	71.8 ± 6.4	74.3 ± 5.9	0.10
e GFR (mL/min, mean ± SD)	62.4 ± 19.5	60.4 ± 18.7	65.2 ± 20.5	0.32
BMI (kg/m^2^, mean ± SD)	28.8 ± 4.9	28.1 ± 4.5	29.2 ± 5.1	0.27
Baseline QRS				
QRS duration (ms, mean ± SD)	108.6 ± 29.2	111.1 ± 29.8	105 ± 28.5	0.38
Normal QRS (*n*, %)	47 (64.4)	27 (62.8)	20 (66.7)	0.80
LBBB (*n*, %)	12 (16.4)	5 (11.6)	7 (23.3)	0.21
RBBB (*n*, %)	14 (19.2)	11 (25.6)	3 (10)	0.13
Echocardiography				
LVEF (%, mean ± SD)	52.5 ± 11.8	51.6 ± 11.3	53.8 ± 12.6	0.43
LA diameter (mm, mean ± SD)	41.1 ± 6.2	43.5 ± 5.9 *	36.8 ± 4.7 *	0.01
RA diameter (mm, mean ± SD)	36.8 ± 6.2	37.9.1 ± 6.4	34.9 ± 5.2	0.10
Comorbidities				
Hypertension (*n*, %)	65 (89)	37 (86)	28 (93.3)	0.46
Diabetes mellitus (*n*, %)	26 (35.6)	16 (37.2)	10 (33.3)	0.80
Ischemic disease (*n*, %)	19 (26)	11 (25.6)	8 (26.7)	1
Renal failure (*n*, %)	29 (39.7)	14 (32.6)	15 (50)	0.15
Persistent AF (*n*, %)	15 (20.5)	9 (20.9)	6 (20)	1
Treatment				
RAAS antagonists (*n*, %)	65 (89)	37 (86)	28 (93.3)	0.46
Beta-blockers (*n*, %)	54 (74)	33 (76.7)	21 (70)	0.59
MRAs (*n*, %)	13 (17.8)	9 (20.9)	4 (13.3)	0.54
Anticoagulants (*n*, %)	31 (42.5)	20 (46.5)	11 (36.7)	0.47

SD, standard deviation; eGFR, estimated glomerular filtration rate; BMI, body mass index; LBBB, left bundle branch block; RBBB, right bundle branch block; LVEF, left ventricular ejection fraction; LA, left atrium; RA, right atrium; RAAS, renin–angiotensin–aldosterone system; MRA, mineral receptors antagonist; *—significantly different from male/female.

**Table 2:** Procedural characteristics.

Procedural Parameters	Overall	Male (*n* = 43)	Female (*n* = 30)	*p*-Value
Baseline QRS duration (ms)	108.6 ± 29.2	111 ± 29.8	105 ± 28.5	0.38
Paced QRS duration (ms)	94.5 ± 19.4	98.9 ± 20.1 *	88.1 ± 16.6 *	0.02
Detection (ms, mean ± SD)	4.1 ± 2.5	4.1 ± 2.3	4.1 ± 2.9	0.94
Pacing threshold (V, mean ± SD)	1.1 ± 0.8	1.1 ± 0.7	1.2 ± 0.9	0.50
Selective capture (*n*, %)	35 (47.9)	20 (46.5)	15 (50)	0.81
Impedance (Ohm, mean ± SD)	465.4 ± 107.5	468.3 ± 104.8	461.3 ± 112.8	0.79
Fluoroscopy time (min, mean ± SD)	8.9 ± 7.5	8.8 ± 7.7	8.9 ± 7.2	0.95

SD—standard deviation; *—significantly different from male/female.

**Table 3:** Uni/multivariate regression analysis of pacing threshold increase by at least 1 V after HBP.

Parameters	HR (95% CI)	*p* Value	HR (95% CI)	*p* Value
Sex	0.69 (0.27–1.77)	0.45		
Age (years)	0.96 (0.89–1.03)	0.26		
Baseline QRS duration (ms)	1.01 (0.99–1.02)	0.49		
Paced QRS duration (ms)	1.02 (1.00–1.04)	0.02	1.02 (1.00–1.05)	0.02
Type of capture	2.3 (0.86–6.00)	0.09	1.89 (0.69–5.20)	0.21
Procedural pacing threshold > 1.25 V	0.67 (0.24–1.86)	0.44		
LV ejection fraction (%)	1.08 (1.01–1.17)	0.03	1.08 (1.02–1.16)	0.01
LA volume (mL)	1.07 (0.96–1.19)	0.21		
RA volume (mL)	1.05 (0.94–1.18)	0.38		
Ischemic disease	0.38 (0.15–0.95)	0.04	0.26 (0.10–0.68)	0.01
Hypertension	0.71 (0.16–3.09)	0.65		
Diabetes Mellitus	1.99 (0.66–6.02)	0.22		
Atrial fibrillation	1.10 (0.72–1.68)	0.66		

HBP, His bundle pacing; HR, hazard ratio; LV, left ventricular; LA, left atrial; RA, right atrial.

**Table 4:** Type of capture and transition with decremental pacing over time.

Initial	Follow-Up	Overall	Male (*n* = 43)	Female (*n* = 30)	*p*-Value
NS-Myo	NS-Myo	32	20	12	0.34
NS-S	NS-S	29	19	10	0.46
NS-Myo	NS-S	5	2	3	0.39
NS-S	NS-Myo	6	1	5	0.07

NS, non-selective; Myo, myocardial; S, selective.

## Data Availability

The datasets are available upon reasonable request to the corresponding author.

## References

[1] de Vries L.M., Dijk W.A., Hooijschuur C.A.M., Leening M.J.G., Stricker B.H.C., van Hemel N.M. (2016). Utilisation of cardiac pacemakers over a 20-year period: Results from a nationwide pacemaker registry. Neth. Hear. J..

[2] Matsubara T., Sumiyoshi M., Kimura A., Minami-Takano A., Maruyama K., Kimura Y., Tabuchi H., Hayashi H., Odagiri F., Sekita G. (2022). Trend in Age at the Initial Pacemaker Implantation in Patients With Bradyarrhythmia—A 50-Year Analysis (1970–2019) in Japan. Circ. J..

[3] Pyatt J.R., Somauroo J.D., Jackson M., Grayson A.D., Osula S., Aggarwal R.K., Charles R.G., Connelly D.T. (2002). Long-term survival after permanent pacemaker implantation: Analysis of predictors for increased mortality. EP Europace.

[4] Nowak B., Misselwitz B., Erdogan A., Funck R., Irnich W., Israel C., Olbrich H.-G., Schmidt H., Sperzel J., Zegelman M. (2009). Do gender differences exist in pacemaker implantation?—results of an obligatory external quality control program. EP Europace.

[5] Kiehl E.L., Makki T., Kumar R., Gumber D., Kwon D.H., Rickard J.W., Kanj M., Wazni O.M., Saliba W.I., Varma N. (2016). Incidence and predictors of right ventricular pacing-induced cardiomyopathy in patients with complete atrioventricular block and preserved left ventricular systolic function. Hear. Rhythm. O2.

[6] Upadhyay G.A., Razminia P., Tung R. (2020). His-bundle pacing is the best approach to physiological pacing. Hear. Rhythm. O2.

[7] Keene D., Anselme F., Burri H., Pérez Ó.C., Čurila K., Derndorfer M., Foley P., Gellér L., Glikson M., Huybrechts W. (2023). Conduction system pacing, a European survey: Insights from clinical practice. EP Europace.

[8] Abdelrahman M., Subzposh F.A., Beer D., Durr B., Naperkowski A., Sun H., Oren J.W., Dandamudi G., Vijayaraman P. (2018). Clinical Outcomes of His Bundle Pacing Compared to Right Ventricular Pacing. J. Am. Coll. Cardiol..

[9] Vinther M., Risum N., Svendsen J.H., Møgelvang R., Philbert B.T. (2021). A Randomized Trial of His Pacing Versus Biventricular Pacing in Symptomatic HF Patients With Left Bundle Branch Block (His-Alternative). JACC Clin. Electrophysiol..

[10] Pestrea C., Cicala E., Gherghina A., Ortan F., Pop D. (2023). Feasibility of Permanent His Bundle Pacing in the Elderly vs the Very Elderly. A Single-Center Mid-Term Follow-Up Study. Clin. Interv. Aging.

[11] Burri H., Jastrzebski M., Cano Ó., Čurila K., de Pooter J., Huang W., Israel C., Joza J., Romero J., Vernooy K. (2023). EHRA clinical consensus statement on conduction system pacing implantation: Endorsed by the Asia Pacific Heart Rhythm Society (APHRS), Canadian Heart Rhythm Society (CHRS), and Latin American Heart Rhythm Society (LAHRS). EP Europace.

[12] Tokavanich N., Prasitlumkum N., Mongkonsritragoon W., Cheungpasitporn W., Thongprayoon C., Vallabhajosyula S., Chokesuwattanaskul R. (2021). A network meta-analysis and systematic review of change in QRS duration after left bundle branch pacing, His bundle pacing, biventricular pacing, or right ventricular pacing in patients requiring permanent pacemaker. Sci. Rep..

[13] Upadhyay G.A., Vijayaraman P., Nayak H.M., Verma N., Dandamudi G., Sharma P.S., Saleem M., Mandrola J., Genovese D., Oren J.W. (2019). On-treatment comparison between corrective His bundle pacing and biventricular pacing for cardiac resynchronization: A secondary analysis of the His-SYNC Pilot Trial. Hear. Rhythm. O2.

[14] Abdin A., Aktaa S., Vukadinović D., Arbelo E., Burri H., Glikson M., Meyer C., Munyombwe T., Nielsen J.C., Ukena C. (2021). Outcomes of conduction system pacing compared to right ventricular pacing as a primary strategy for treating bradyarrhythmia: Systematic review and meta-analysis. Clin. Res. Cardiol..

[15] Kawashima T., Sasaki H. (2005). A macroscopic anatomical investigation of atrioventricular bundle locational variation relative to the membranous part of the ventricular septum in elderly human hearts. Surg. Radiol. Anat..

[16] Keene D., Arnold A.D., Jastrzębski M., Burri H., Zweibel S., Crespo E., Chandrasekaran B., Bassi S., Joghetaei N., Swift M. (2019). His bundle pacing, learning curve, procedure characteristics, safety, and feasibility: Insights from a large international observational study. J. Cardiovasc. Electrophysiol..

[17] Vijayaraman P., Patel N., Colburn S., Beer D., Naperkowski A., Subzposh F.A. (2022). His-Purkinje Conduction System Pacing in Atrioventricular Block: New Insights into Site of Conduction Block. JACC Clin. Electrophysiol..

[18] Zanon F., Abdelrahman M., Marcantoni L., Naperkowski A., A Subzposh F., Pastore G., Baracca E., Boaretto G., Raffagnato P., Tiribello A. (2019). Long term performance and safety of His bundle pacing: A multicenter experience. J. Cardiovasc. Electrophysiol..

[19] Beer D., A Subzposh F., Colburn S., Naperkowski A., Vijayaraman P. (2020). His bundle pacing capture threshold stability during long-term follow-up and correlation with lead slack. EP Europace.

[20] Wu S., Shang W., Ye Y., Su L., Wang S., Cai M., Wang D., He Y., Zheng R., Fu G. (2024). Sex differences outcomes in conduction system pacing for patients with typical left bundle branch block. Int. J. Cardiol..

[21] Stangl D., Buia V., Walascheck J., Rittger H., Bastian D., Vitali-Serdoz L. (2024). The pursuit of gender equality: A substudy of the PACE-CONDUCT trial on gender differences in His bundle pacing implantation. EP Europace.

[22] Upadhyay G.A., Sun W., Nayak H.M., Aziz Z., Beaser A.D., Ozcan C., Tung R. (2021). B-PO01-040 Lead Stability in His Bundle Pacing—Incidence, Predictors, and Timing of Increased Pacing Thresholds. Hear. Rhythm. O2.

